# Systemic lupus erythematosus with acrocyanosis after AstraZeneca COVID‐19 vaccination

**DOI:** 10.1002/kjm2.12630

**Published:** 2022-11-30

**Authors:** Huei‐Jing Wang, Yng Sun, Cheng‐Che Eric Lan

**Affiliations:** ^1^ Department of Dermatology Kaohsiung Medical University Hospital, Kaohsiung Medical University Kaohsiung Taiwan; ^2^ Department of Dermatology, College of Medicine Kaohsiung Medical University Kaohsiung Taiwan

As newly developed COVID‐19 vaccines were employed for controlling disease dissemination, few cases with new onset of SLE after mRNA‐based vaccination were reported.^1^, ^2^ Here, we present a patient of newly diagnosed SLE with acrocyanosis after receiving one dose of AstraZeneca COVID‐19 vaccine, an adenovirus‐based DNA vaccine.

A 37‐year‐old woman presented with a 1‐week history of painful cyanosis over right index finger after receiving her first dose of AstraZeneca COVID‐19 vaccine 2 weeks ago. No other discomfort including rash elsewhere were noted, and her medical history was unremarkable. At the clinic, laboratory investigations revealed neutropenia (total white blood cell count 3850/mm^3^), elevation of D‐dimer (0.65 mg/L fibrinogen equivalent unit), reduced protein S level (46%), and positive anti‐nuclear antibodies (titer 1:160). Despite the use of aspirin (100 mg daily) and pentoxifylline (800 mg daily), cyanotic fingers continued to progress. As thrombotic events were reported to develop after AstraZeneca COVID‐19 vaccine, she was admitted for further evaluation and treatment. Upon admission, physical examination showed cyanosis with tenderness over distal portion of right index finger (Figure 1A). Laboratory tests revealed low C3 (85.7 mg/dl). Autoimmune panel showed elevated antibodies against dsDNA (71 IU/ml), ribonucleoprotein (32 EliAU/ml), and Phosphatidylserine/Prothrombin complex (IgG and IgM were 126.093 and 65.236 units). Angiography was done, which showed intermittent vessel spasms on digital arteries without any evidence of thrombosis. Skin biopsy on right index fingertip only showed dilated vessels and scanty inflammatory infiltrates (Figure 1C) with no signs of vasculitis or thrombosis and no remarkable finding of direct immunofluorescence staining. According to the classification criteria from European Alliance of Associations for Rheumatology/American College of Rheumatology (EULAR/ACR), a diagnosis of systemic lupus erythematosus (SLE) manifest with acrocyanosis was made (a score of 12). The patient received hydrocortisone (200 mg daily) and hydroxychloroquine (400 mg daily). Nifedipine (30 mg daily) was also added for vasospasm related cyanosis, and the treatment resulted in increased blood flow of the fingers as measured by laser Doppler flowmetry. Steroid dose was gradually tapered after improvement (Figure 1B). Currently, the patient is on maintenance treatment consisting of hydroxychloroquine treatment (200 mg twice daily).

**FIGURE 1:**
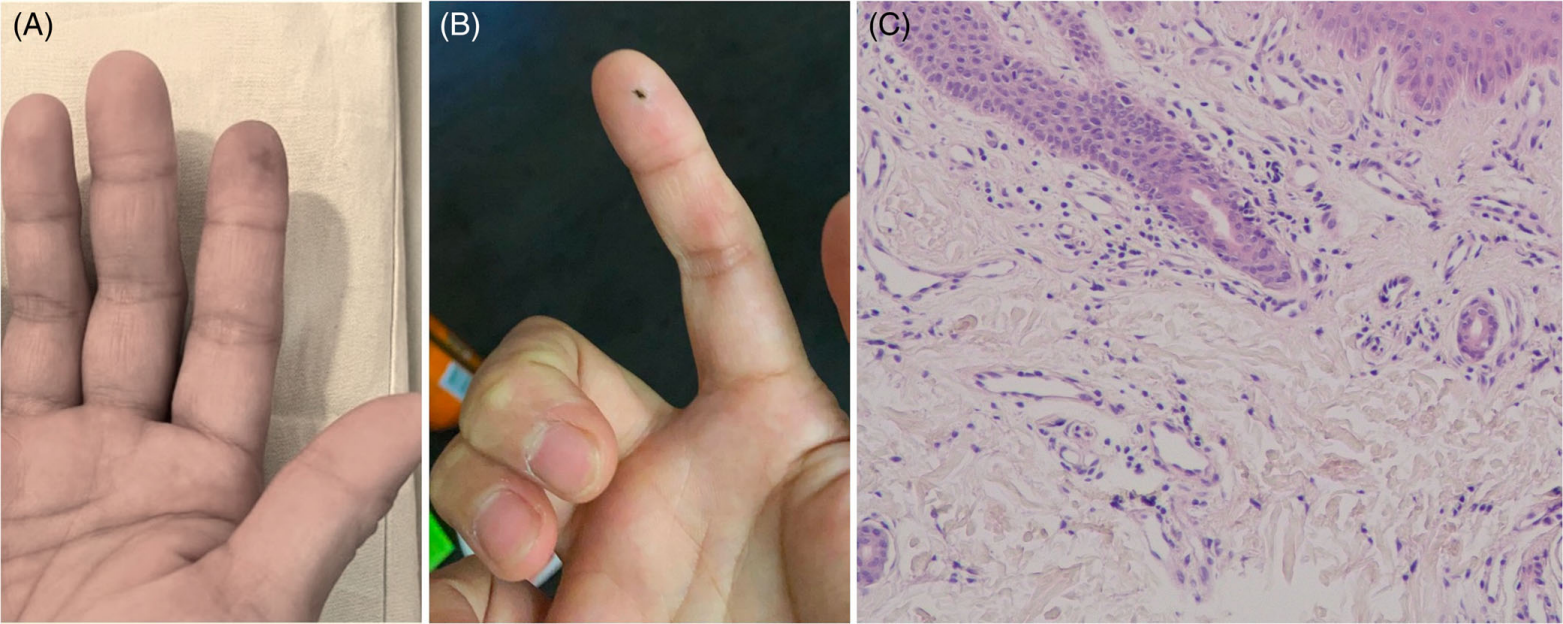
The pathology and therapeutic outcome of fingertip lesion before and after treatment. The right index fingertip showed cyanosis before treatment (A). (B) denotes a fully recovery of cyanosis with a post‐biopsy wound after 25 days follow‐up with treatment. Pathology of cyanotic fingertip showed dilated vessels with scanty inflammatory infiltrates (C).

Here, we present a patient of newly diagnosed SLE with acrocyanosis after receiving one dose of AstraZeneca COVID‐19 vaccine. SLE is an autoimmune disease with multisystem involvement, associated with autoantibodies against various autoantigens, including dsDNA. Previous research has suggested that SLE and COVID‐19 vaccination share similar mechanism including type 1 interferon and proinflammatory cytokine pathways. RNA virus and RNA vaccines may trigger Toll‐like receptors (TLR) 7/8, inducing type 1 interferons production.^3^ Similar effects are also noted with DNA virus and DNA virus‐derived vaccine, via TLR9 and cyclic GMP‐AMP (cGAS) and the cyclic GMP‐AMP receptor stimulator of interferon genes (STING) pathway.^4^ To date, only one other report demonstrated a possible causality with adenovirus‐based DNA vaccine and SLE.^5^

In summary, we present a case of newly onset of SLE after receiving adenovirus‐based DNA COVID‐19 vaccine. Timely diagnosis and early intervention may lead to appropriate treatment strategy.

The patient's family have given written informed consent to publication.

## CONFLICT OF INTEREST

All authors declare no conflict of interest.
